# Generation of path-polarization hyperentanglement using quasi-phase-matching in quasi-periodic nonlinear photonic crystal

**DOI:** 10.1038/s41598-017-05271-7

**Published:** 2017-07-10

**Authors:** Guangqiang He, Chengrui Zhu, Yao Jiang, Jie Ren, Ying Guo, Jietai Jing

**Affiliations:** 1 State Key Laboratory of Advanced Optical Communication Systems and Networks, Electronic Engineering Department, Shanghai Jiao Tong University, Shanghai, 200240 China; 2 State Key Laboratory of Precision Spectroscopy, East China Normal University, Shanghai, 200062 China; 3 School of Information Science and Engineering, Central South University, Changsha, 410083 China

## Abstract

A compact scheme for the generation of path-polarization entangled photon pairs is proposed by using a quasi-periodic nonlinear photonic crystal to simultaneously accomplish four spontaneous parametric down-conversion processes. Moreover, we report experimental scheme to measure the polarization entanglement and path entanglement separately and theoretically get numerical results that verify some predictions about the hyperentanglement. This method can be expanded for the generation of multi-partite and two-photon path-polarization hyperentanglement in a single quasi-periodic nonlinear photonic crystal structure. This compact quantum light source can be used as a significant ingredient in quantum information science.

## Introduction

Entanglement plays a key role in the applications of quantum information science such as quantum cryptography^1^, quantum teleportation^2^ and dense coding^3^. Consequently, to create and manipulate entanglement using an integrated quantum light source has been a defining experimental goal in recent years.

One solid strategy resorts to quasi-phase matching (QPM) of spontaneous parametric down-conversion (SPDC) processes^4^ in a designed nonlinear photonic crystal (NPC) since it avoids bulky and complex experimental elements. This method has been applied to create some kinds of entanglements such as two-photon polarization entanglement^5^, single-photon entanglement^6^, path entanglement^7^, *etc*. However, the NPCs used in these schemes are periodic. One limitation is that they are usually used to phase match only processes whose mismatch vectors are integer multiples of a single vector (in 1D case) or a vectorial sum of only two base vectors (in 2D case). Consequently, a single periodic NPC structure is not usually used to simultaneously phase match multiple SPDCs. So we adopt the engineering of quasi-periodic NPCs^8, 9^, which provides greater design flexibility for phase matching several different SPDCs and thus provides more possibilities in entanglement generation.

We are inspired by the notion of hyperentanglement^10^, which refers to the entanglement at multiple degrees of freedom (DOFs) such as polarization, frequency, energy time, *etc*. Specifically, we focus on producing hyperentanglement at the polarization and spatial mode by using a single designed quasi-periodic NPC to phase match several SPDCs. This method not only incorporates the many applications of path-entanglement including quantum precise phase measurement^11^, super-resolution quantum lithography^11^, and encoding of multilevel systems in spatial mode of single photon^12, 13^, but also expands the Hilbert space, thereby provides advantages in many parts of quantum information science such as enlarging the channel capacity in super dense coding^14^, enhancing the security of quantum crytography^15, 16^, and assisting complete Bell-state discrimination^17, 18^. Moreover, theoretically, this method enables to create multiple spatial modes (larger than two) in the path-polarization hyperentanglement in a single quasi-periodic NPC instead of using different cascaded periodic NPCs as in some generation schemes of path entanglement^7^. So this method can be seen as a more compact scheme.

This paper is arranged as follows. In results, we describe the generation of path-polarization hyperentangled photon pairs by using QPM of 4 SPDC processes in a designed quasi-period NPC. The design parameters of the NPC, its structure and its Fourier transform are given. The experimental setup is given which incorporates Hong-Ou-Mandel quantum inference measurements^19, 20^. As to this setup, numerical simulation results are given which verify our predictions about the hyperentanglement. In discussion, we discuss how the basic model can be expanded for the generation of multi-partite and two-photon path-polarization hyperentanglement. In method, we introduce the principle of designing the crystal.

## Results

### Generation of path-polarization hyperentangled photon pairs

**Figure 1:**
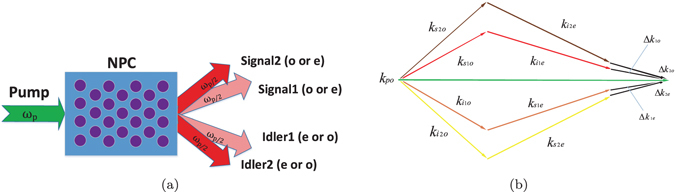
(**a**) Schematic for the generation of path-polarization hyperentangled photon pairs. NPC is designed to qusi-phase-matching 4 SPDC processes. The pump photon with frequency of *ω*_*p*_ is injected into the designed NPC and gets through either of the 4 SPDC processes. Consequently, in our engineering, the path-polarization hyperentangled signal and idler photon with frequency of *ω*_*p*_/2 should be emitted from the NPC. (**b**) QPM condition for the 4 SPDC processes in generation of path-polarization hyperentangled photon pairs.

The schematic for the generation of path-polarization hyperentangled photon pairs is displayed in Fig. 1. We have a pump photon with the frequency of *ω*_*p*_ injected into the designed NPC—in which it will get through either of the 4 SPDC processes— and the signal and idler photons with frequency *ω*_*p*_/2 are assumed to be generated in our engineering. From an intuitive perspective, the signal and idler photons are firstly polarization entangled; and since they come out from either of the two spatial modes shown in Fig. 1(a), they are also path entangled.

The NPC displayed in Fig. 1(a) is designed to simultaneously accomplish QPM of the 4 different SPDC processes. The QPM condition can be depicted by Fig. 1(b). **k**_*po*_ represents the wave vector of the pump light (*o* light); ${{\bf{k}}}_{{s}_{1}o(e)}$, ${{\bf{k}}}_{{i}_{1}e(o)}$, ${{\bf{k}}}_{{s}_{2}o(e)}$ and ${{\bf{k}}}_{{i}_{2}e(o)}$ represents the wave vectors of Signal1 (*o* light or *e* light), Idler1 (*e* light or *o* light), Signal2 (*o* light or *e* light) and Idler2 (*e* light or *o* light) respectively. The mismatch vectors are described as

1 $$\begin{array}{rcl}{\rm{\Delta }}{{\bf{k}}}_{1o(e)} & = & {{\bf{k}}}_{po}-{{\bf{k}}}_{{s}_{1}o(e)}-{{\bf{k}}}_{{i}_{1}e(o)}\\ {\rm{\Delta }}{{\bf{k}}}_{2o(e)} & = & {{\bf{k}}}_{po}-{{\bf{k}}}_{{s}_{2}o(e)}-{{\bf{k}}}_{{i}_{2}e(o)}\end{array}$$

To illustrate our design method we have proposed, we are going to take an example with specific parameter values. It must be noted that, these specific parameter values are just used to justify our theory in calculation, maybe the values are not suitable for a realistic case. However, if necessary, we can design the lattice with appropriate parameter values in any realistic case, including wavelength, temperature, directions of the wave vectors and so on. Thus there is no loss of generality.

we consider a very typical laser, Nd:YAG laser, whose wavelength is 532 nm. Now we set the wavelength of the pump light as 532 *nm* and that of the signal and idler light is 1064 nm. The direction of the wave vectors of beams ${{\bf{k}}}_{po},\,{{\bf{k}}}_{{s}_{1}o(e)},\,{{\bf{k}}}_{{i}_{1}e(o)},\,{{\bf{k}}}_{{s}_{2}o(e)},\,{{\bf{k}}}_{{i}_{2}e(o)}$ are 0°, 58°, −58°, 74°, −74° respectively. Periodically poled lithium niobat (PPLN) is chosen as the NPC material and the working temperature is 21 °C. We adopt sellmeier equations under this condition^21^ and figure out the mismatch vectors as

2 $$\begin{array}{rcl}{\rm{\Delta }}{{\bf{k}}}_{1e} & = & \mathrm{(13.708},\mathrm{0.384)}\mu {m}^{-1}\\ {\rm{\Delta }}{{\bf{k}}}_{1o} & = & \mathrm{(13.708},-\mathrm{0.384)}\mu {m}^{-1}\\ {\rm{\Delta }}{{\bf{k}}}_{2e} & = & \mathrm{(20.296},\mathrm{0.435)}\mu {m}^{-1}\\ {\rm{\Delta }}{{\bf{k}}}_{2o} & = & \mathrm{(20.296},-\mathrm{0.435)}\mu {m}^{-1}\end{array}$$

**Figure 2:**
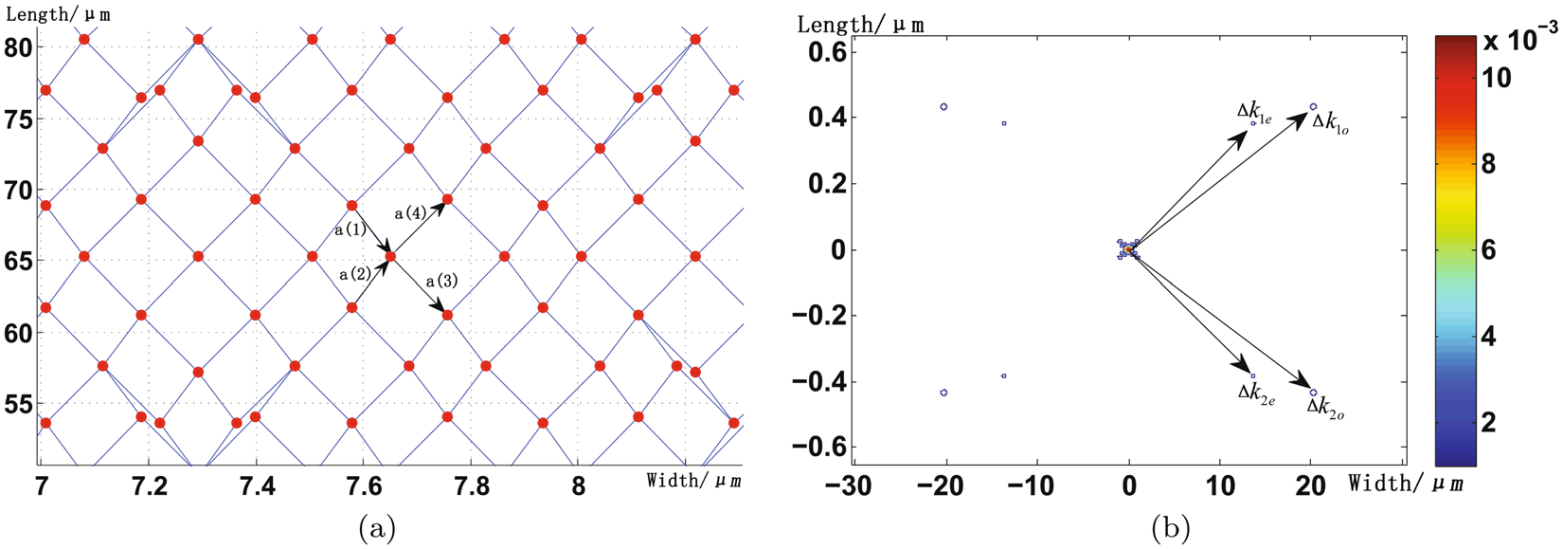
(**a**) Structure of the PPLN NPC. Scale is in *μm*, drawn with an aspect ratio of approximately **1:25**. The arrows indicate the four tiling vectors *a* ^(*i*)^ (i = 1, 2, 3, 4). Note that for clarity the PPLN NPC structure is partially depicted and the total area is 50 *μm* × 700 *μm*. (b) Fourier transform ***G***(*k*) of the **PPLN NPC**. Scale is in *μm* ^−1^. The arrows indicate the mismatch vectors ${\rm{\Delta }}{{\bf{k}}}_{1e},{\rm{\Delta }}{{\bf{k}}}_{1o},{\rm{\Delta }}{{\bf{k}}}_{2e},{\rm{\Delta }}{{\bf{k}}}_{2o}$.

Through engineering of the PPLN NPC89 to accomplish QPM of the mismatch vectors, the structure of PPLN NPC is depicted by Fig. 2(a) and the tiling vectors shown in Fig. 2(a) are

3 $$\begin{array}{rcl}{{\bf{a}}}^{\mathrm{(1)}} & = & \mathrm{(7.87},-\mathrm{46.67)}\mu m\\ {{\bf{a}}}^{\mathrm{(2)}} & = & \mathrm{(7.87},\mathrm{46.67)}\mu m\\ {{\bf{a}}}^{\mathrm{(3)}} & = & \mathrm{(17.33},-\mathrm{55.03)}\mu m\\ {{\bf{a}}}^{\mathrm{(4)}} & = & \mathrm{(17.33},\mathrm{55.03)}\mu m\end{array}$$

Each red dot with radius of 1 *μm* in Fig. 2(a) is called motif^9^. Figure 2(a) actually depicts the distribution of nonlinear coefficient *χ* ^(2)^ in the PPLN NPC, which is obtained by the convolution between the quasi-periodic lattice and motif. In the motif (red dot) *χ* ^(2)^ = 1 while *χ* ^(2)^ = −1 in other areas of the PPLN NPC. We can also express the PPLN NPC structure mathematically as $g({\bf{r}})=a({\bf{r}})\times (u({\bf{r}})\otimes s({\bf{r}}))$, where a(**r**) denotes the total area of the PPLN NPC, *u*(**r**) is a sum of delta functions and denotes the lattice function, *s*(**r**) denotes the motif function, $\otimes$ is convolution operator. The Fourier transform of the PPLN NPC determines the conversion efficiency of the SPDC processes and can be written as refs 8 and 9

4 $$\begin{array}{rcl}G({\bf{k}}) & = & FT\{g({\bf{r}})\}=U({\bf{k}})\otimes A({\bf{k}})\times S({\bf{k}})={\rm{\Delta }}\chi (U({\bf{k}})\otimes {\int }_{a({\bf{r}})}{e}^{i{\bf{k}}\cdot {\bf{r}}}{d}^{2}r){\int }_{s({\bf{r}})}{e}^{i{\bf{k}}\cdot {\bf{r}}}{d}^{2}r\\  & = & 2SA{\rm{\Delta }}\chi \frac{{J}_{1}(kR)}{kR}\{U({\bf{k}})\otimes [sinc(\frac{{k}_{x}{L}_{x}}{2})sinc(\frac{{k}_{y}{L}_{y}}{2})]\},\end{array}$$

where $k=|{\bf{k}}|$, *k*_*x*_, *k*_*y*_ indicate the *x* and *y* components of **k**, ${\rm{\Delta }}\chi$ is the absolute difference between the positive and negative values used for ${\chi }^{\mathrm{(2)}}$, *J*_1_ is the first Bessel function, *S* is a circle of radius $R=1\,\mu m$, *A* is a rectangle of sides *L*_*x*_ × *L*_*y*_—which indicates the total area of PPLN NPC (*L*_*x*_ = 0.5 *mm*, *L*_*y*_ = 2.5 *mm* in our engineering), *U*(**k**) is the Fourier transform of lattice function $u({\bf{r}})$ and is the sum of delta functions. Figure 2(b) depicts the Fourier transform of the PPLN NPC. We can clearly distinguish Bragg peaks at the positions of the required mismatch vectors ${\rm{\Delta }}{{\bf{k}}}_{1e},{\rm{\Delta }}{{\bf{k}}}_{1o},{\rm{\Delta }}{{\bf{k}}}_{2e},{\rm{\Delta }}{{\bf{k}}}_{2o}$, while there are no Bragg peaks at unwanted positions—which shows desirable conversion efficiency of SPDC processes in this PPLN NPC. Near the required mismatch vectors, the Fourier transform can also be written as

5 $$\begin{array}{l}G({\bf{k}})={G}_{je(o)}({\bf{k}}-{\rm{\Delta }}{{\bf{k}}}_{je(o)})={G}_{je(o)}({\rm{\Delta }}{\bf{k}})=2SA{\rm{\Delta }}\chi \frac{{J}_{1}({\rm{\Delta }}{{\bf{k}}}_{je(o)}R)}{{\rm{\Delta }}{{\bf{k}}}_{je(o)}R}[U({\rm{\Delta }}{{\bf{k}}}_{je(o)})sinc(\frac{{\rm{\Delta }}{{\bf{k}}}_{x}{L}_{x}}{2})sinc(\frac{{\rm{\Delta }}{{\bf{k}}}_{y}{L}_{y}}{2})],\end{array}$$

where $j$ = 1, 2, ${\rm{\Delta }}{\bf{k}}={\bf{k}}-{\rm{\Delta }}{{\bf{k}}}_{je(o)}$, ${\rm{\Delta }}{{\bf{k}}}_{je(o)}=|{\rm{\Delta }}{{\bf{k}}}_{je(o)}|$, ${\rm{\Delta }}{{\bf{k}}}_{x}$ and ${\rm{\Delta }}{{\bf{k}}}_{y}$ indicate the *x* and *y* components of Δ*k*. Note here that if more spatial modes are introduced–which implies more SPDC processes to achieve–we can prevent the decrease of SPDC efficiency by promoting the size of our designed PPLN

Under the first-order perturbation approximation^22^, through the QPM of 4 SPDC processes in the designed PPLN NPC, the two-photon state can be written as

6 $$|\psi \rangle ={\alpha }_{0}\sum _{k\mathrm{=1}}^{2}\int d\upsilon [{\varphi }_{eo}^{k}(\upsilon ){\hat{a}}_{{s}_{k}e}^{\dagger }(\frac{{\omega }_{p}}{2}+\upsilon ){\hat{a}}_{{i}_{k}o}^{\dagger }(\frac{{\omega }_{p}}{2}-\upsilon )|0\rangle +{\varphi }_{oe}^{k}(\upsilon ){\hat{a}}_{{s}_{k}o}^{\dagger }(\frac{{\omega }_{p}}{2}+\upsilon ){\hat{a}}_{{i}_{k}e}^{\dagger }(\frac{{\omega }_{p}}{2}-\upsilon )|0\rangle ],$$

*α*_0_ in the equation is a normalization constant. Subscript *eo* and *oe* indicate the polarization of the signal and idler photons. Subscript *k* represents the spatial mode. The numerical two-photon mode function ${\varphi }_{eo}^{k}(\upsilon )$ and ${\varphi }_{oe}^{k}(\upsilon )$ can be obtained from the Fourier transform of the PPLN NPC near the required mismatch vectors which is expressed by Eq. (5) (${\varphi }_{eo}^{k}(\upsilon )={G}_{ke}({\rm{\Delta }}{\bf{k}}),{\varphi }_{oe}^{k}(\upsilon )={G}_{ko}({\rm{\Delta }}{\bf{k}})$). And the relationship between the detuning frequency $\upsilon$ and Δ**k** is

7 $$\begin{array}{rcl}{\rm{\Delta }}{{\bf{k}}}_{x} & = & \upsilon [\frac{\cos \,{\theta }_{{s}_{k}e(o)}}{{u}_{ge(o)}}-\frac{\cos \,{\theta }_{{i}_{k}o(e)}}{{u}_{go(e)}}]+\frac{{\upsilon }^{2}}{2}[\cos \,{\theta }_{{s}_{k}e(o)}\frac{d}{d\omega }(\frac{1}{{u}_{e(o)}}){|}_{\omega =\frac{{\omega }_{p}}{2}}\\  &  & +\cos \,{\theta }_{{i}_{k}o(e)}\frac{d}{d\omega }(\frac{1}{{u}_{o(e)}}){|}_{\omega =\frac{{\omega }_{p}}{2}}],\\ {\rm{\Delta }}{{\bf{k}}}_{y} & = & \upsilon [\frac{\sin \,{\theta }_{{s}_{k}e(o)}}{{u}_{ge(o)}}-\frac{\sin \,{\theta }_{{i}_{k}o(e)}}{{u}_{go(e)}}]+\frac{{\upsilon }^{2}}{2}[\sin \,{\theta }_{{s}_{k}e(o)}\frac{d}{d\omega }(\frac{1}{{u}_{e(o)}}){|}_{\omega =\frac{{\omega }_{p}}{2}}\\  &  & +\sin \,{\theta }_{{i}_{k}o(e)}\frac{d}{d\omega }(\frac{1}{{u}_{o(e)}}){|}_{\omega =\frac{{\omega }_{p}}{2}}],\end{array}$$

where ${\theta }_{{s}_{k}e(o)}$ indicates the angle between ${{\bf{k}}}_{{s}_{k}e(o)}$ and $x$ axis, ${\theta }_{{i}_{k}o(e)}$ indicates the angle between ${{\bf{k}}}_{{i}_{k}o(e)}$ and *x* axis, ${u}_{ge(o)}=\frac{d\omega }{d{k}_{e(o)}}{|}_{\omega ={\omega }_{p}\mathrm{/2}}$ is group velocity of the signal or idler ($e$ light or $o$ light). Equation 6 can be simplified and written as

8 $$|\psi \rangle =({C}_{eo}^{1}|HV\rangle +{C}_{oe}^{1}|VH\rangle ){|1\rangle }_{{s}_{1}}{|1\rangle }_{{i}_{1}}{|0\rangle }_{{s}_{2}}{|0\rangle }_{{i}_{2}}+({C}_{eo}^{2}|HV\rangle +{C}_{oe}^{2}|VH\rangle ){|0\rangle }_{{s}_{1}}{|0\rangle }_{{i}_{1}}{|1\rangle }_{{s}_{2}}{|1\rangle }_{{i}_{2}},$$

where $${C}_{eo}^{1}={C}_{oe}^{1}=0.483,{C}_{eo}^{2}={C}_{oe}^{2}=0.516$$, and $${({C}_{eo}^{1})}^{2}+{({C}_{oe}^{1})}^{2}+{({C}_{eo}^{2})}^{2}+{({C}_{oe}^{2})}^{2}=1$$.

This is aptly the required path-polarization hyperentanglement. Note that it is significant to match the efficiency of SPDC processes of different spatial modes because it will achieve maximally entangled states. However to date, there is no general design methods available to achieve this condition which means that this condition can only be achieved in some selected cases of us.

**Figure 3:**
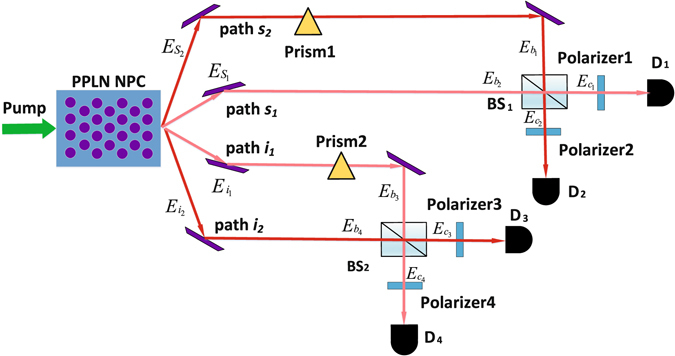
Experimental setup which is used to verify path and polarization entanglement. *BS*_1_ and *BS*_2_ are beam splitters with transmission coefficient *T* = 0.5. To verify path and polarization entanglement, we collect coincidence counting from detectors while adjusting prisms and polarizers.

We design an experimental scheme and the criterions^23^ to verify the path and polariztion entanglement separately. The experiment setup is shown in Fig. 3.

In Fig. 3, ${\hat{E}}_{{s}_{k}}$ and ${\hat{E}}_{{i}_{k}}$ (*k*=1, 2) stand for the signal and idler light fields generated from SPDC processes in the PPLN NPC. They are expressed as

9 $${\hat{E}}_{{s}_{k}}({t}_{{s}_{k}})=\frac{1}{\sqrt{2\pi }}\int d\upsilon ({\varphi }_{eo}^{k}(\upsilon ){\hat{a}}_{{s}_{k}e}^{\dagger }({\omega }_{p}\mathrm{/2}+\upsilon )+{\varphi }_{oe}^{k}(\upsilon ){\hat{a}}_{{s}_{k}o}^{\dagger }({\omega }_{p}\mathrm{/2}+\upsilon )){e}^{-i({\omega }_{p}\mathrm{/2}+\upsilon ){t}_{{s}_{k}}},$$

10 $${\hat{E}}_{{i}_{k}}({t}_{{i}_{k}})=\frac{1}{\sqrt{2\pi }}\int d\upsilon ({\varphi }_{eo}^{k}(\upsilon ){\hat{a}}_{{i}_{k}e}^{\dagger }({\omega }_{p}\mathrm{/2}-\upsilon )+{\varphi }_{oe}^{k}(\upsilon ){\hat{a}}_{{i}_{k}o}^{\dagger }({\omega }_{p}\mathrm{/2}-\upsilon )){e}^{-i({\omega }_{p}\mathrm{/2}-\upsilon ){t}_{{i}_{k}}}\mathrm{.}$$

The phase relation, *β*_1_ between *s*_1_, *s*_2_ and *β*_2_ between *i*_1_, *i*_2_, can be set by tilting two prisms. And we have ${\hat{E}}_{{b}_{1}}={e}^{i{\beta }_{1}}{\hat{E}}_{{s}_{2}}$, ${\hat{E}}_{{b}_{2}}={\hat{E}}_{{s}_{1}}$, ${\hat{E}}_{{b}_{3}}={e}^{i{\beta }_{2}}{\hat{E}}_{{i}_{1}}$, ${\hat{E}}_{{b}_{4}}={\hat{E}}_{{i}_{2}}$. Generally, in Heisenberg picture, the evolution of operators in a BS is expressed as

11 $${\hat{E}}_{out}^{\mathrm{(1)}}=\sqrt{T}{\hat{E}}_{in}^{\mathrm{(1)}}+\sqrt{R}{\hat{E}}_{in}^{\mathrm{(2)}},$$

12 $${\hat{E}}_{out}^{\mathrm{(2)}}=\sqrt{T}{\hat{E}}_{in}^{\mathrm{(2)}}-\sqrt{R}{\hat{E}}_{in}^{\mathrm{(1)}}\mathrm{.}$$

The evolution of operators in BS1 and BS2 can be expressed as

13 $${\hat{E}}_{{c}_{1}}=\frac{1}{\sqrt{2}}({\hat{E}}_{{b}_{1}}+{\hat{E}}_{{b}_{2}}),$$

14 $${\hat{E}}_{{c}_{2}}=\frac{1}{\sqrt{2}}({\hat{E}}_{{b}_{1}}-{\hat{E}}_{{b}_{2}}),$$

15 $${\hat{E}}_{{c}_{3}}=\frac{1}{\sqrt{2}}({\hat{E}}_{{b}_{3}}+{\hat{E}}_{{b}_{4}}),$$

16 $${\hat{E}}_{{c}_{4}}=\frac{1}{\sqrt{2}}({\hat{E}}_{{b}_{3}}-{\hat{E}}_{{b}_{4}}\mathrm{).}$$

where *j* = 1, 2. Without loss of generality, we show theoretically the detected result at *D*_1_, *D*_2_ after classical interference at BS1 and *D*_3_, *D*_4_ after classical interference at BS2. The polarizers are temporarily removed so that all polarization components are included. To verify the path entanglement, the coincidence count of detectors *D*_1_ and *D*_3_ is measured, which is proportional to the expected value of the opeartor ${\hat{E}}_{{c}_{3}}({t}_{i}){\hat{E}}_{{c}_{1}}({t}_{s}){\hat{E}}_{{c}_{1}}^{\dagger }({t}_{s}){\hat{E}}_{{c}_{3}}^{\dagger }({t}_{i})$.

17 $$\langle {\hat{E}}_{{c}_{3}}({t}_{i}){\hat{E}}_{{c}_{1}}({t}_{s}){\hat{E}}_{{c}_{1}}^{\dagger }({t}_{s}){\hat{E}}_{{c}_{3}}^{\dagger }({t}_{i})\rangle =\langle \psi |{\hat{E}}_{{c}_{3}}({t}_{s}){\hat{E}}_{{c}_{1}}({t}_{i}){\hat{E}}_{{c}_{1}}^{\dagger }({t}_{s}){\hat{E}}_{{c}_{3}}^{\dagger }({t}_{i})|\psi \rangle ={T}_{1}+{T}_{2}+{T}_{3}+{T}_{4},$$

where

18 $$\begin{array}{rcl}{T}_{1} & = & \frac{1}{16{\pi }^{2}}{\alpha }_{0}^{2}\int {(d\upsilon )}^{4}{[{({\varphi }_{eo}^{1}(\upsilon ))}^{3}+{({\varphi }_{oe}^{1}(\upsilon ))}^{3}]}^{2},\\ {T}_{2} & = & \frac{1}{16{\pi }^{2}}{\alpha }_{0}^{2}\int {(d\upsilon )}^{4}{[{({\varphi }_{eo}^{2}(\upsilon ))}^{3}+{({\varphi }_{oe}^{2}(\upsilon ))}^{3}]}^{2},\\ {T}_{3} & = & \frac{1}{16{\pi }^{2}}{\alpha }_{0}^{2}\int {(d\upsilon )}^{4}[{({\varphi }_{eo}^{1}(\upsilon ))}^{3}+{({\varphi }_{oe}^{1}(\upsilon ))}^{3}]\\  &  & \times [{({\varphi }_{eo}^{2}(\upsilon ))}^{3}+{({\varphi }_{oe}^{2}(\upsilon ))}^{3}]{e}^{i({\omega }_{p}\mathrm{/2}+\upsilon )({t}_{{s}_{1}}-{t}_{{s}_{2}}+2{\beta }_{1}/{\omega }_{p})+i({\omega }_{p}\mathrm{/2}-\upsilon )({t}_{{i}_{1}}-{t}_{{i}_{2}}-2{\beta }_{2}/{\omega }_{p})},\\ {T}_{4} & = & \frac{1}{16{\pi }^{2}}{\alpha }_{0}^{2}\int {(d\upsilon )}^{4}[{({\varphi }_{eo}^{1}(\upsilon ))}^{3}+{({\varphi }_{oe}^{1}(\upsilon ))}^{3}]\\  &  & \times [{({\varphi }_{eo}^{2}(\upsilon ))}^{3}+{({\varphi }_{oe}^{2}(\upsilon ))}^{3}]{e}^{-i({\omega }_{p}\mathrm{/2}+\upsilon )({t}_{{s}_{1}}-{t}_{{s}_{2}}+2{\beta }_{1}/{\omega }_{p})-i({\omega }_{p}\mathrm{/2}-\upsilon )({t}_{{i}_{1}}-{t}_{{i}_{2}}-2{\beta }_{2}/{\omega }_{p})}\mathrm{.}\end{array}$$

Here we introduce a phase difference ${\rm{\Delta }}{t}_{s}={t}_{{s}_{1}}-{t}_{{s}_{2}}$, ${\rm{\Delta }}{t}_{i}={t}_{{i}_{1}}-{t}_{{i}_{2}}$. After submitting the numerical two-photon mode function to Eq. (18), approximately we have

19 $$C({c}_{1},{c}_{3})\propto 1.02+\,\cos ({\omega }_{p}\mathrm{/2}\cdot ({\rm{\Delta }}{t}_{s}-{\rm{\Delta }}{t}_{i})+{\beta }_{1}-{\beta }_{2}\mathrm{).}$$

We can get coincidence counts of (*D*_1_, *D*_4_), (*D*_2_, *D*_3_) and (*D*_2_, *D*_4_) by the same method. The expected coincidence count can be written as

20 $$C({c}_{1},{c}_{4})\propto 1.02-\,\cos ({\omega }_{p}\mathrm{/2}\cdot ({\rm{\Delta }}{t}_{s}-{\rm{\Delta }}{t}_{i})+{\beta }_{1}-{\beta }_{2}),$$

21 $$C({c}_{2},{c}_{3})\propto 1.02-\,\cos ({\omega }_{p}\mathrm{/2}\cdot ({\rm{\Delta }}{t}_{s}-{\rm{\Delta }}{t}_{i})+{\beta }_{1}-{\beta }_{2}),$$

22 $$C({c}_{2},{c}_{4})\propto 1.02+\,\cos ({\omega }_{p}\mathrm{/2}\cdot ({\rm{\Delta }}{t}_{s}-{\rm{\Delta }}{t}_{i})+{\beta }_{1}-{\beta }_{2}\mathrm{).}$$

The entangled state $|\psi \rangle$ can be adoped to test the violation of a Bell inequality^24^ with ${\rm{\Delta }}{t}_{s}={\rm{\Delta }}{t}_{i}=0$. The parameter ${S}_{k}$ is expressed as

23 $${S}_{k}=|E({\beta }_{1},{\beta }_{2})-E({\beta }_{1},{{\beta }_{2}}^{\ast })+E({{\beta }_{1}}^{\ast },{\beta }_{2})+E({{\beta }_{1}}^{\ast },{{\beta }_{2}}^{\ast }\mathrm{)|.}$$

where

24 $$E({\beta }_{1},{\beta }_{2})=\frac{C({c}_{1},{c}_{3})+C({c}_{2},{c}_{4})-C({c}_{1},{c}_{4})-C({c}_{2},{c}_{3})}{C({c}_{1},{c}_{3})+C({c}_{2},{c}_{4})+C({c}_{1},{c}_{4})+C({c}_{2},{c}_{3})}\mathrm{.}$$

Two photon coincidence can be measured by using the phase setting *β*_1_ = 0, ${{\beta }_{1}}^{\ast }=\frac{\pi }{2}$ and ${\beta }_{2}=\frac{\pi }{4}$, ${{\beta }_{2}}^{\ast }=\frac{3\pi }{4}$. The expected value, ${S}_{k}=2.357 > 2$, verifies the path entanglement between *s*_1_, *i*_1_ and *s*_2_, *i*_2_.

Then we discuss the measurement of polarization entanglement^25^. The evolution of operators in Polarizer1 and Polarizer2 can be written as

25 $${\hat{E}}_{{c}_{1}}^{\text{'}}={\hat{E}}_{{c}_{1}(e)}\,\cos ({\theta }_{1})+{\hat{E}}_{{c}_{1}(o)}\,\sin ({\theta }_{1}),$$

26 $${\hat{E}}_{{c}_{3}}^{\text{'}}={\hat{E}}_{{c}_{3}(e)}\,\cos ({\theta }_{2})+{\hat{E}}_{{c}_{3}(o)}\,\sin ({\theta }_{2}\mathrm{).}$$

where ${\hat{E}}_{{c}_{k}(e)}$, ${\hat{E}}_{{c}_{k}(o)}$ (*k* = 1, 3) indicate the orthonormal polarization components of ${\hat{E}}_{{c}_{k}}$. To verify the polarization entanglement, the Coincidence Counting of detectors *D*_1_ and *D*_3_ is measured, which is proportional to the expected value of the operator ${\hat{E}}_{{c}_{3}}^{\text{'}}({t}_{i}){\hat{E}}_{{c}_{1}}^{\text{'}}({t}_{s}){\hat{E}}_{{c}_{1}}^{\dagger ^{\prime} }({t}_{s}){\hat{E}}_{{c}_{3}}^{\dagger ^{\prime} }({t}_{i})$. We have

27 $$\langle {\hat{E}}_{{c}_{3}}^{\text{'}}({t}_{i}){\hat{E}}_{{c}_{1}}^{\text{'}}({t}_{s}){\hat{E}}_{{c}_{1}}^{\dagger ^{\prime} }({t}_{s}){\hat{E}}_{{c}_{3}}^{\dagger ^{\prime} }({t}_{i})\rangle =\langle \psi |{\hat{E}}_{{c}_{3}}^{\text{'}}({t}_{s}){\hat{E}}_{{c}_{1}}^{\text{'}}({t}_{i}){\hat{E}}_{{c}_{1}}^{\dagger ^{\prime} }({t}_{s}){\hat{E}}_{{c}_{3}}^{\dagger ^{\prime} }({t}_{i})|\psi \rangle ={W}_{1}+{W}_{2}+{W}_{3}+{W}_{4},$$

where

28 $$\begin{array}{c}{W}_{1}=\frac{1}{16{\pi }^{2}}{\alpha }_{0}^{2}\int {(d\upsilon )}^{4}{[({\varphi }_{eo}^{1}(\upsilon {))}^{3}\cos ({\theta }_{1})\sin ({\theta }_{2})+{({\varphi }_{oe}^{1}(\upsilon ))}^{3}\sin ({\theta }_{1})\cos ({\theta }_{2})]}^{2},\\ {W}_{2}=\frac{1}{16{\pi }^{2}}{\alpha }_{0}^{2}\int {(d\upsilon )}^{4}{[({\varphi }_{eo}^{2}(\upsilon {))}^{3}\cos ({\theta }_{1})\sin ({\theta }_{2})+{({\varphi }_{oe}^{2}(\upsilon ))}^{3}\sin ({\theta }_{1})\cos ({\theta }_{2})]}^{2},\\ {W}_{3}=\frac{1}{16{\pi }^{2}}{\alpha }_{0}^{2}\int {(d\upsilon )}^{4}[{({\varphi }_{eo}^{1}(\upsilon ))}^{3}\,\cos ({\theta }_{1})\sin ({\theta }_{2})+{({\varphi }_{oe}^{1}(\upsilon ))}^{3}\,\sin ({\theta }_{1})\cos ({\theta }_{2})]\\ \quad \,\quad \times [({\varphi }_{eo}^{2}(\upsilon {))}^{3}\,\cos ({\theta }_{1})\sin ({\theta }_{2})+{({\varphi }_{oe}^{2}(\upsilon ))}^{3}\,\sin ({\theta }_{1})\cos ({\theta }_{2})]\\ \quad \,\quad \times {e}^{i({\omega }_{p}\mathrm{/2}+\upsilon )({\rm{\Delta }}{t}_{s}+2{\beta }_{1}/{\omega }_{p})+i({\omega }_{p}\mathrm{/2}-\upsilon )({\rm{\Delta }}{t}_{i}+2{\beta }_{2}/{\omega }_{p})},\\ {W}_{4}=\frac{1}{16{\pi }^{2}}{\alpha }_{0}^{2}\int {(d\upsilon )}^{4}[{({\varphi }_{eo}^{1}(\upsilon ))}^{3}\,\cos ({\theta }_{1})\sin ({\theta }_{2})+{({\varphi }_{oe}^{1}(\upsilon ))}^{3}\,\sin ({\theta }_{1})\cos ({\theta }_{2})]\\ \quad \,\quad \times [({\varphi }_{eo}^{2}(\upsilon {))}^{3}\,\cos ({\theta }_{1})\sin ({\theta }_{2})+{({\varphi }_{oe}^{2}(\upsilon ))}^{3}\,\sin ({\theta }_{1})\cos ({\theta }_{2})]\\ \quad \,\quad \times {e}^{-i({\omega }_{p}\mathrm{/2}+\upsilon )({\rm{\Delta }}{t}_{s}+2{\beta }_{1}/{\omega }_{p})-i({\omega }_{p}\mathrm{/2}-\upsilon )({\rm{\Delta }}{t}_{i}+2{\beta }_{2}/{\omega }_{p})}\mathrm{.}\end{array}$$

Submit the numerical two-photon mode function, the result can be expressed as

29 $$\begin{array}{l}\langle {\hat{E}}_{{c}_{3}}^{\text{'}}({t}_{i}){\hat{E}}_{{c}_{1}}^{\text{'}}({t}_{s}){\hat{E}}_{{c}_{1}}^{\dagger ^{\prime} }({t}_{s}){\hat{E}}_{{c}_{3}}^{\dagger ^{\prime} }({t}_{i})\rangle \propto [1.02+\,\cos ({\omega }_{p}/2\cdot ({\rm{\Delta }}{t}_{s}+{\rm{\Delta }}{t}_{i})+{\beta }_{1}+{\beta }_{2})]\,{\sin }^{2}({\theta }_{1}+{\theta }_{2}\mathrm{).}\end{array}$$

**Figure 4:**
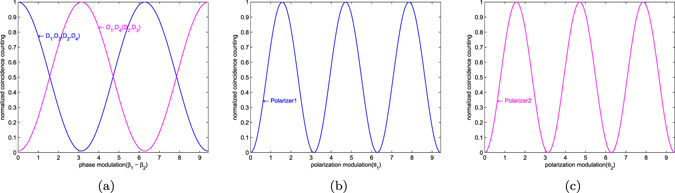
(a) Coincidence counting of *D*_1_, *D*_3_(*D*_2_, *D*_4_) and *D*_1_, *D*_4_ (*D*_2_, *D*_3_) with respect to phase modulation(*β*_1_–*β*_2_). (b) Coincidence counting of *D*_1_ and *D*_3_ with respect to modulation of Polarizer1 (*θ*_1_). (c) Coincidence counting of *D*_1_ and *D*_3_ with respect to modulation of Polarizer2 (*θ*_2_).

Note here that the effect of Prism1 and Prism2 (*β*_1_ and *β*_2_) can be used to compensate for the phase difference between the signal photons (Δ*t*_*s*_) and that between idler photons (Δ*t*_*i*_). Specifically, we need to adjust Prism1(Prism2) until the classical interference at BS1(BS2) results in a peak detected intensity at *D*_1_ (*D*_2_). After phase difference compensating, when ${\theta }_{2}({\theta }_{1})=0$, the relation between Coincidence Counting of *D*_1_, *D*_2_ and *θ*_1_(*θ*_2_) can be depicted by Fig. 4(a)—in which the interference fringes with visibility of 100% prove the polarization entanglement. We use ${\rm{\Delta }}{\gamma }_{1}$ and ${\rm{\Delta }}{\gamma }_{2}$ to represent transmission errors of BS1 and BS2. Moreover, if transmission error of BS is taken into account, we have the transmission coefficient of BS1 to be $\sin (\pi \mathrm{/4}+{\rm{\Delta }}{\gamma }_{1})$, and that of BS2 to be $\sin (\pi \mathrm{/4}+{\rm{\Delta }}{\gamma }_{2})$. Consequently, ${W}_{1}^{^{\prime} }=2{\cos }^{2}(\pi \mathrm{/4}+{\rm{\Delta }}{\gamma }_{1})\,{\sin }^{2}(\pi \mathrm{/4}+{\rm{\Delta }}{\gamma }_{2}){W}_{1}$, ${W}_{2}^{^{\prime} }=2\,{\cos }^{2}(\pi \mathrm{/4}+{\rm{\Delta }}{\gamma }_{2})\,{\sin }^{2}(\pi \mathrm{/4}+{\rm{\Delta }}{\gamma }_{1}){W}_{2}$, ${W}_{3}^{^{\prime} }=2\,\cos (\pi /4+{\rm{\Delta }}{\gamma }_{1})\,\cos (\pi /4+{\rm{\Delta }}{\gamma }_{2})\,\sin (\pi /4+{\rm{\Delta }}{\gamma }_{1})\,\sin (\pi /4+{\rm{\Delta }}{\gamma }_{2}){W}_{3}$, ${W}_{4}^{^{\prime} }=2\,\cos (\pi /4+{\rm{\Delta }}{\gamma }_{1})\,\cos (\pi /4+{\rm{\Delta }}{\gamma }_{2})$ $\sin (\pi /4+{\rm{\Delta }}{\gamma }_{1})\,\sin (\pi /4+{\rm{\Delta }}{\gamma }_{2}){W}_{4}$. ${W}_{1}$ and ${W}_{2}$ (${W}_{3}$ and ${W}_{4}$) are affected by the same manner.

Figure 4(b) and Fig. 4(c) depict the relation between normalized coincidence counting and angular settings of polarizers and shows the visibility, *V* = 1. Two photon coincidence counting can be measured by using the angular setting *θ*_1_ = 0, ${{\theta }_{1}}^{\ast }=\frac{\pi }{4}$ and ${\theta }_{2}=\frac{\pi }{8}$, ${{\theta }_{2}}^{\ast }=\frac{5\pi }{8}$. The expected value, ${S}_{k}=2.828 > 2$, verifies the polarization entanglement between signal and idler.

## Discussion

### **Multi-partite and two-photon path-polarization hyperentanglement**

**Figure 5:**
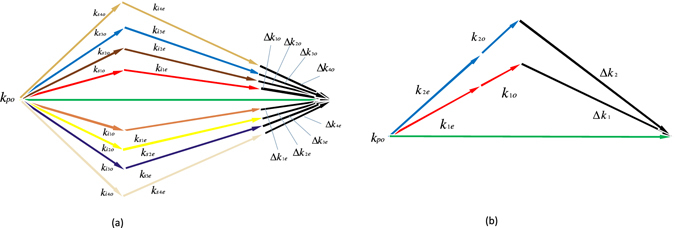
(**a**) The QPM condition for 8-partite path-polarization hyperentanglement. (**b**) The QPM condition for two-photon path-polarization hyperentanglement.

Equation (8) illustrates the proposed path-polarization hyperentanglement. This is a basic 4-SPDC model and some adjustments to the NPC engineering will give new forms to the hyperentanglement. For example, if the engineering of NPC incorparates 8 SPDC processes instead of 4, the QPM condition can be illustrated by Fig. 5(a). And the result is that the number of spatial modes becomes 4 instead of 2, which implicates that the path entanglement part in the hyperentanglement changes from 4-partite to 8-partite. It can be described as

30 $$\begin{array}{rcl}|\psi \rangle  & = & ({C}_{eo}^{1}|HV\rangle +{C}_{oe}^{1}|VH\rangle ){|1\rangle }_{{s}_{1}}{|1\rangle }_{{i}_{1}}{|0\rangle }_{{s}_{2}}{|0\rangle }_{{i}_{2}}{|0\rangle }_{{s}_{3}}{|0\rangle }_{{i}_{3}}{|0\rangle }_{{s}_{4}}{|0\rangle }_{{i}_{4}}\\  &  & +({C}_{eo}^{2}|HV\rangle +{C}_{oe}^{2}|VH\rangle ){|0\rangle }_{{s}_{1}}{|0\rangle }_{{i}_{1}}{|1\rangle }_{{s}_{2}}{|1\rangle }_{{i}_{2}}{|0\rangle }_{{s}_{3}}{|0\rangle }_{{i}_{3}}{|0\rangle }_{{s}_{4}}{|0\rangle }_{{i}_{4}}\\  &  & +({C}_{eo}^{3}|HV\rangle +{C}_{oe}^{3}|VH\rangle ){|0\rangle }_{{s}_{1}}{|0\rangle }_{{i}_{1}}{|0\rangle }_{{s}_{2}}{|0\rangle }_{{i}_{2}}{|1\rangle }_{{s}_{3}}{|1\rangle }_{{i}_{3}}{|0\rangle }_{{s}_{4}}{|0\rangle }_{{i}_{4}}\\  &  & +({C}_{eo}^{4}|HV\rangle +{C}_{oe}^{4}|VH\rangle ){|0\rangle }_{{s}_{1}}{|0\rangle }_{{i}_{1}}{|0\rangle }_{{s}_{2}}{|0\rangle }_{{i}_{2}}{|0\rangle }_{{s}_{3}}{|0\rangle }_{{i}_{3}}{|1\rangle }_{{s}_{4}}{|1\rangle }_{{i}_{4}}\mathrm{.}\end{array}$$

Theoretically, if multiple paths are established, multi-partite path-polarization entanglement can be generated. Moreover, if there are still 4 designed SPDC processes but the signal and idler are designed to be emitted from the same path, the QPM condition can be illustrated by Fig. 5(b).

## Method

Designing a proper crystal is a key point for phase matching 4 SPDC processes. A general method to design frequency converters that will phase match any set of interacting waves is provided by the so-called generalized dual grid method (DGM)^9^. In this method, a dual structure, called the dual grid, which contains all the topological information required to built the quasi-crystal is first constructed. Then, using a simple transformation, this dual grid is transformed to a quasi-crystal. The Dual Grid Method can be adapted to match different processes. For different processes, the only thing you need to do is changing the mismatch vectors. Moreover, the Dual Grid Method could be implemented by a computer program, which is convenient to design a crystal.
